# 2834. Comparative Effectiveness of Oral Antibiotics to Treat Urinary Tract Infections in Male and Female Outpatients

**DOI:** 10.1093/ofid/ofad500.2444

**Published:** 2023-11-27

**Authors:** Karl Madaras-Kelly, Jeremy Boyd, Laura Bond

**Affiliations:** Idaho State University, Boise, ID; Boise VA Medical Center, Boise, Idaho; Boise VA medical Center/Boise State University, Boise, Idaho

## Abstract

**Background:**

Professional and regulatory agency guidelines recommend that fluoroquinolones not be used as first-line therapy to treat uncomplicated UTI. Increasingly, β-lactams are being used to treat UTI despite limited evidence supporting their effectiveness. The study aim was to compare the efficacy of β-lactams and other commonly prescribed antibiotics for UTI to fluoroquinolones.

**Methods:**

A multi-centered retrospective cohort study of outpatient visits with a UTI diagnosis between 2019-2021 was developed. Inclusion criteria: In-person visit in the Emergency Department or Primary/Urgent Care with ICD-10 documented UTI diagnosis and oral antibiotic prescription filled within 0-2 d. Exclusion criteria: Prior UTI < 30d, co-diagnosis where antibiotics were indicated, hospital discharge < 7d, scheduled urological procedure < 7d, pregnancy. Outcome: UTI related admission or outpatient return visit within 3-30d. Demographic, co-morbidity, vitals, laboratory, and prescription data were extracted. Propensity scores were used to balance treatments and GEE models were used to estimate outcomes.

**Results:**

There were 73,334 visits in 130 VA medical centers. Mean (S.D.) cohort age was 66.0 (15.8) y, 22.9% were female. Antibiotics prescribed N (%): β-lactams (cephalosporins, aminopenicillins) 27,385 (37.3%), nitrofurantoin 15,509 (21.1%), trimethoprim / sulfamethoxazole (TMP/SMX) 15,453 (21.1%), and fluoroquinolones 14,997 (20.4%). The overall 30-day clinical failure rate was 10,525 (14.4%).

Comparative Effectiveness of Oral Antibiotics for Outpatient UTIs

**Figure:**
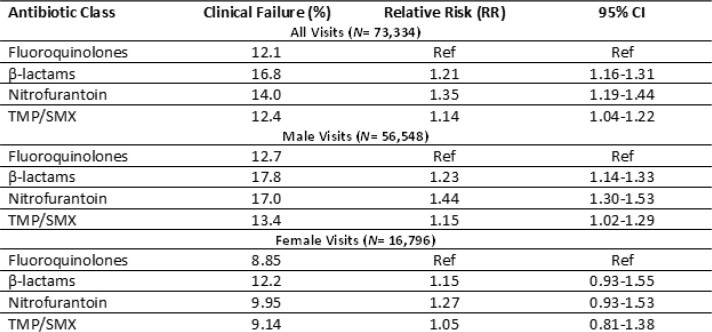

**Conclusion:**

Commonly prescribed antibiotics for outpatient UTI were associated with a modest increase in 30-day clinical failure relative to fluoroquinolones. Effectiveness differences were most pronounced in male patients. Clinicians should carefully differentiate between complicated and uncomplicated UTI in males and consider preferentially prescribing fluoroquinolones.

**Disclosures:**

**Karl Madaras-Kelly, PharmD, MPH**, BioMerioux: Grant/Research Support

